# Atopic Dermatitis: Molecular Alterations between Lesional and Non-Lesional Skin Determined Noninvasively by In Vivo Confocal Raman Microspectroscopy

**DOI:** 10.3390/ijms241914636

**Published:** 2023-09-27

**Authors:** Michael Zolotas, Johannes Schleusener, Jürgen Lademann, Martina C. Meinke, Georgios Kokolakis, Maxim E. Darvin

**Affiliations:** 1 Center of Experimental and Applied Cutaneous Physiology, Department of Dermatology, Venereology and Allergology, Charité—Universitätsmedizin Berlin, Corporate Member of Freie Universität Berlin and Humboldt-Universität zu Berlin, Charitéplatz 1, 10117 Berlin, Germany; 2 Psoriasis Research and Treatment Centre, Department of Dermatology, Venereology and Allergology, Charité—Universitätsmedizin Berlin, Corporate Member of Freie Universität Berlin and Humboldt-Universität zu Berlin, Charitéplatz 1, 10117 Berlin, Germany

**Keywords:** atopic eczema, atopic dermatitis, skin barrier function, stratum corneum, lipid ordering, keratin structure, natural moisturising factor, water binding, skin hydration, carotenoids

## Abstract

Atopic dermatitis (AD)/atopic eczema is a chronic relapsing inflammatory skin disease affecting nearly 14% of the adult population. An important pathogenetic pillar in AD is the disrupted skin barrier function (SBF). The atopic stratum corneum (SC) has been examined using several methods, including Raman microspectroscopy, yet so far, there is no depth-dependent analysis over the entire SC thickness. Therefore, we recruited 21 AD patients (9 female, 12 male) and compared the lesional (LAS) with non-lesional atopic skin (nLAS) in vivo with confocal Raman microspectroscopy. Our results demonstrated decreased total intercellular lipid and carotenoid concentrations, as well as a shift towards decreased orthorhombic lateral lipid organisation in LAS. Further, we observed a lower concentration of natural moisturising factor (NMF) and a trend towards increased strongly bound and decreased weakly bound water in LAS. Finally, LAS showed an altered secondary and tertiary keratin structure, demonstrating a more folded keratin state than nLAS. The obtained results are discussed in comparison with healthy skin and yield detailed insights into the atopic SC structure. LAS clearly shows molecular alterations at certain SC depths compared with nLAS which imply a reduced SBF. A thorough understanding of these alterations provides useful information on the aetiology of AD and for the development/control of targeted topical therapies.

## 1. Introduction

Atopic dermatitis (AD) is a chronic relapsing inflammatory skin disease that affects 15–30% of children and nearly 14% of adults [1,2], inflicting a heavy disease burden [3]. AD is one of the diseases of the atopic march together with bronchial asthma, allergic rhinitis, and type-I allergies [4], and its clinical manifestations include symptoms such as dry skin, indurated eczematous lesions with erythema and scaling, as well as pruritus, causing excoriation and, in the long term, skin thickening and lichenification [4,5,6].

Even though the exact pathogenesis of AD is not completely understood, it is assumed to be a combination of (epi)genetic (e.g., filaggrin mutations), immunologic (inflammation with a T_H_2-shift and higher IgE-levels), and exogenous factors, such as exposure to allergens and irritants, infections and alteration in the physiological skin microbiome, excessive hygiene, and climatic changes [5,7,8,9,10,11,12,13]. One of the defining pathophysiologic aspects of AD is the disruption of the skin barrier function (SBF), correlating with the disease severity [5,14]. The skin barrier dysfunction—partly attributable to genetic factors—is thought to interplay with a dysregulated immune system, resulting in inadequate response to external factors, prolonged wound healing, and higher allergic sensitisation [8,9,15,16,17]. A detailed understanding of the atopic SBF is required to thoroughly understand the disease’s pathogenesis.

The main actor regarding the SBF is the epidermis and especially its most superficial layer, the stratum corneum (SC), accounting for most of the skin’s functionality [18,19]. The SC consists of denucleated corneocytes, which represent the end product of keratinisation [20] and are strongly interconnected with the help of corneodesmosomes, crosslinked cornified envelopes, including the proteins involucrin, loricrin, and filaggrin, and keratin-filaggrin attachments [21]. They provide the main barrier against mechanical and chemical disturbances [4]. In what is often described as the “brick and mortar model” of the SC [18,22], the corneocytes are imbedded in an intercellular lipid (ICL) matrix, consisting mainly of ceramides, cholesterol, and free fatty acids (FFA) [23], which is essential in maintaining an intact SBF (especially regarding the regulation of water diffusion) [4]. The ICL organisation in the SC directly influences the SBF: orthorhombic lateral and *trans*-conformation-shifted lamellar organisations promote a denser packing of ICL and intact SBF [24,25] nonhomogeneous throughout the SC. The highest SBF is observed at a depth of ≈20–40% SC thickness [26]. A further relevant fraction of the lipophilic substances in the human SC are carotenoids, consisting mostly of *β*-carotene and lycopene, facilitating the orthorhombic organisation of ICL [27] and playing a major antioxidative and photoprotective role in the SC [28]. Also important in maintaining an intact SBF are the natural moisturising factor (NMF) molecules derived from filaggrin, which correspond to certain highly humectant amino acids, maintaining the SC’s hydration, acidic pH, adequate maturation and healthy microbiologic milieu by reducing colonisation with *Staphylococcus aureus* [8,29].

The impaired SBF in AD is a result of both structural and functional alterations in the SC [8,9] and is not limited to lesional atopic skin (LAS) sites, as it is well established that the entire atopic skin, including non-lesional (nLAS), has a higher permeability for hydrophilic and lipophilic chemicals compared with healthy skin (HS) [30]. Furthermore, the SC of LAS is known to be thinner than the SC of HS [31]. The transepidermal water loss (TEWL), as a measure of SBF [18], is increased in AD [6,9], with differences being observed between LAS and nLAS as well as between nLAS and HS [32,33]. Knor et al. [34] even described differences between lesional, perilesional, non-lesional atopic, and HS regarding pH (with highest values in LAS) and hydration (with lowest values in LAS) of the SC. Matsuki et al. found a correlation between AD severity and TEWL increase and, to a lesser extent, SC hydration [35], and a similar correlation is also found for the higher pH [36], which is thought to influence lipid metabolism, serine proteases (SPs) activity, and vulnerability to infections [8]. An increased activity of SPs, also associated with lower SC hydration [31], is generally considered a reason for reduced SBF in AD [16,31], disturbing the physiologic lipid metabolism (e.g., reducing the total amount of lipids in the SC) and protein balance, that are essential for the SBF [8,9], and even promoting T_H_2-shifted inflammation by increasing IL-1*α* and IL-1*β* [15].

Further well-examined SBF-related factors in AD are filaggrin loss-of-function-mutations because of the important structural function of filaggrin in HS and its role in producing components of the natural moisturising factor (NMF) (especially at superficial SC depth), with consequential impact on the physiologic hydration, (acidic) pH and SP activity regulation, and the antimicrobial properties of the SC [4,16]. Thus, it is not surprising that reduced or even total loss of filaggrin in AD is associated with lower NMF concentration, higher TEWL, skin xerosis [4,37], and higher *Staphylococcus aureus* colonisation [29]. While genetic filaggrin mutations are not found in all AD patients [38,39,40], inflammatory cytokines such as IL-4/13 are also able to reduce filaggrin expression. Other described factors responsible for the impaired atopic SBF are dysfunctional tight junctions [41], reduced antimicrobial peptides [4], and a disturbed milieu of commensal microbiome [42,43,44], e.g., with reduced presence of physiologically appearing *Cutibacterium*, *Streptococcus*, *Corynebacterium*, and *Proteobacteria*, in favour of *Staphylococcus* colonisation [45] which correlates with higher TEWL [46]. Finally, important structural aberrations in the atopic SC are observed with respect to the ICL. The total amount of ICL and the number of ceramides (especially ceramides 1 and 3) are found to be lower in atopic skin, and the FFA chain length is reduced in comparison with HS [33,47] with effects on the SBF [9] that will be further discussed below.

Various noninvasive methods have been used to study the alterations in the SC in AD in vivo so far, for example, TEWL measurement, capacitance–conductance determination, reflectance spectroscopy, optical coherence tomography, or diverse methods of lipid analysis [48]. Confocal Raman microspectroscopy (CRM) describes the combination of Raman spectroscopy (i.e., the spectroscopic recording of Stokes Raman-scattered photons after excitation of a probe, e.g., with continuous-wave laser light) and confocal microscopy, which offers the potential of axial resolution into a probe. CRM offers a valuable noninvasive optical method to examine the SC structure and the SBF-related parameters on the molecular level, as it detects specific molecules, their conformation, structure, and concentration with high chemical specificity and sensitivity in in vivo skin studies [49,50]. CRM has been widely used in the past to examine HS [51,52,53,54], the effects of ageing [55], and psoriatic skin [56,57,58]. So far, CRM has already been applied in AD to study the impact of filaggrin mutations [59,60,61], to compare sensitive skin with atopic and non-sensitive skin [62], to compare HS with lesional atopic and psoriatic skin [63], and to create an objective classification score for AD [64]. Janssens et al. have focussed on the lipid analysis of atopic skin [65], and Verzeaux et al. studied several molecular SBF-related parameters comparing LAS and HS up to a depth of 12.5 μm in the SC [66].

Considering the limited analogous investigations of the SC in AD patients on a molecular level, in this study, we compare for the first time the entire SC of LAS with nLAS in vivo in a noninvasive depth-dependent manner using CRM. We apply the processing methods developed in our group [26,67,68], focusing on molecular parameters related to the SBF, such as ICL concentration and organisation, NMF concentration, secondary and tertiary structure of keratin, as well as water mobility states and total water concentration. This will reveal important information regarding the pathophysiology of AD and potentially provide a useful diagnostic tool for disease development and treatment control in the future.

## 2. Results

### 2.1. SC Thickness

Boundaries of the SC could successfully be computed with the method described under Section 4.3 for 43 out of 71 Raman profiles of LAS (≈61%) and for 63 out of 78 profiles of nLAS (≈81%) (Supplementary Tables S1 and S2). The remaining spectra, for which a SC thickness could not be determined, were not considered for further analysis. With a mean SC thickness of 24 ± 5 μm, LAS was found not to differ significantly (*p* = 0.15) from nLAS, with 23 ± 4 μm.

### 2.2. Concentration and Organisation of Intercellular Lipids

**Figure 1:**
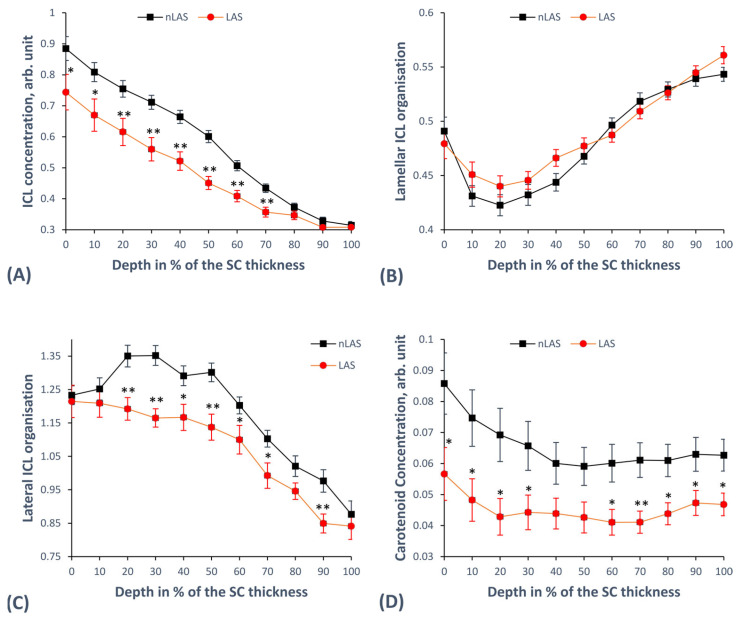
The depth-dependent lipid-related parameters in the SC of LAS vs. nLAS. (**A**) The concentration of ICL given by (*I*_2880_ + *I*_2850_)/*I*_2930_. (**B**) The lamellar organisation of ICL given by *I*_1080_/(*I*_1130_ + *I*_1060_). (**C**) The lateral organisation of ICL given by *I*_2880_/*I*_2850_ upon exclusion of the interference of keratin (the procedure is shown in detail in [26]). (**D**) The concentration of carotenoids given by *I*_1524_. The results are derived from 43 Raman profiles for LAS and 63 Raman profiles for nLAS in total. [*—*p* < 0.05, **—*p* < 0.01 for significant differences between LAS and nLAS; *I*_x_—intensity at Raman peak position “x” (in cm^−1^); ICL—intercellular lipids; SC—stratum corneum; (n)LAS—non-lesional atopic skin, the error bars show the standard error of the means].

In order to obtain the concentration of ICL, we added the decomposed Raman band intensities at 2850 and 2880 cm^−1^ related to ICL and normalised them to the decomposed band intensity at 2930 cm^−1^ related to keratin [53]. The result shows a lower ICL concentration in LAS than nLAS for the superficial and intermediate SC depth (significant for 0–10% and highly significant for 20–70% SC depth, Figure 1A).

Regarding the lamellar organisation of the ICL, we computed the *I*_1080_/(*I*_1130_ + *I*_1060_) ratio, revealing the ratio of *gauche* conformers (associated with the 1080 cm^−1^ band) to all-*trans* conformers (associated with the 1130 and 1060 cm^−1^ bands) [26]. Figure 1B shows similar curves for LAS and nLAS, with a clear tendency towards a higher number of *gauche* conformers in LAS than nLAS at the 10–40% SC depth, which suggests a possibility of impaired SBF in LAS compared with nLAS at this depth. None of the differences reached the level of statistical significance.

Further, we observed the lateral ICL organisation with the help of the ratio of lipid-related band intensities at 2880 to 2850 cm^−1^ (separated from the superposition of keratin), which is sensitive to the orthorhombic and hexagonal lateral packing of the ICL [26]. The ratio is lower, indicating reduced SBF for LAS over most of the SC depth, revealing the presence of a less dense hexagonal organisation, with the strongest deviation from the nLAS curve appearing at 20–50% SC depth. The differences are significant at 40, 60, and 70% and highly significant at 20–30, 50, and 90% SC depth, as shown in Figure 1C.

Finally, we examined the carotenoids, such as *β*-carotene and lycopene, which are the major carotenoids in the human SC [28] and play an important role in the formation of the orthorhombic organisation of ICL [27]. Their concentration was determined using the carotenoid-related Raman band intensity at 1524 cm^−1^ (*I*_1524_) [69]. Figure 1D shows the resulting curves, with LAS clearly having a lower carotenoid concentration than nLAS over the entire SC depth. The differences are significant at 0–30, 60, and 80–100% and highly significant at 70% SC depth.

### 2.3. Concentration of Natural Moisturising Factor (NMF) Molecules

**Figure 2:**
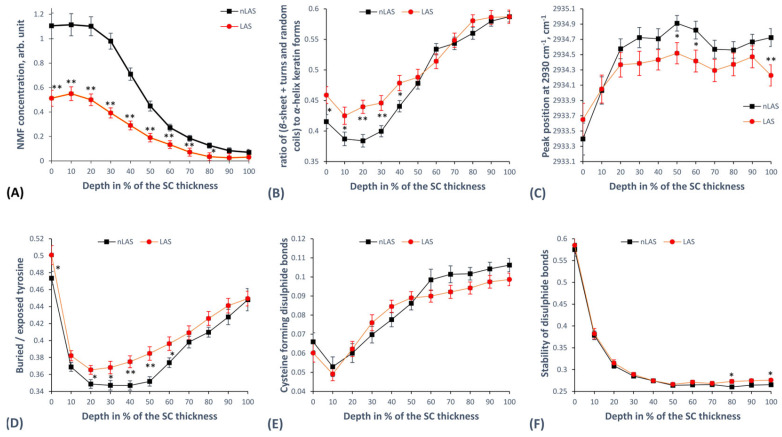
The concentration of NMF (**A**) and parameters of secondary (**B**) and tertiary (**C**–**F**) keratin structure in the SC of LAS vs. nLAS. (**A**) The NMF concentration calculated in a semiquantitative manner with a least-squares fitting method from Skin Tools Software 2.0. (**B**) The ratio of (*β*-sheets, turns, and random coils) to *α*-helices, as a defining parameter of the secondary keratin structure, calculated by (*I*_1670_ + *I*_1685_)/*I*_1655_. (**C**) The shifting of the *I*_2930_ band indicating the folding status of the tertiary keratin structure. (**D**) The ratio of buried to exposed tyrosine rings in side chains of keratin calculated by *I*_830_/*I*_850_. (**E**) The cysteine forming disulphide bonds in keratin calculated by *I*_690–712_/*I*_474–578_. (**F**) Stability of the disulphide bonds calculated by *I*_474–508_/*I*_474–578_ showing the ratio of the most energetically stable to all possible conformations of the disulphide bonds in keratin filaments. The results are derived from 43 Raman profiles for LAS and 63 Raman profiles for nLAS in total. In the case of NMF in (**A**), 43 Raman profiles for LAS and 58 Raman profiles for nLAS were used. [*—*p* < 0.05, **—*p* < 0.01 for significant differences between LAS and nLAS; *I*_x_—intensity at Raman peak position “x” (in cm^−1^); NMF—natural moisturising factor; SC—stratum corneum; (n)LAS—non-lesional atopic skin, the error bars show the standard error of the means].

In total, 43 LAS and 58 nLAS profiles were used for statistical analysis after the exclusion of profiles for which either NMF or SC thickness could not be successfully computed. The NMF consists of the combined concentrations of alanine, serine, glycine, proline, ornithine, histidine at pH4 and pH7, and pyrrolidone carboxylic acid (Figure 2A). It could be seen that LAS has a much lower NMF concentration than nLAS, with the difference being highest at the superficial SC depth. For 0–70%, the difference of the means is highly significant, and for 80% SC depth, is significant.

### 2.4. Keratin Structure

#### 2.4.1. Secondary Keratin Structure

The different components of the secondary structure of keratin are *α*-helices, *β*-sheets, turns, and random coils. While the *α*-helices provide a highly stable coiled-coil structure, allowing very little interaction with water or other molecules, the other mentioned secondary conformations allow a higher number of interactions with surrounding molecules [68,70]. As an indicator for the stability of the secondary structure, we used the (*I*_1670_ + *I*_1685_)/*I*_1655_ quotient, which reveals the ratio of *β*-sheets, turns, and random coils to *α*-helices [53,71]. This ratio is higher for LAS in the superficial half of the SC depth than nLAS, thus showing a lower prevalence of *α*-helices and a less stable secondary structure, as shown in Figure 2B. The differences are significant for 0–10 and 40% and highly significant for 20–30% SC depth. In the remaining 50–100% SC depth, the curves seem to overlap, indicating no difference.

#### 2.4.2. Tertiary Keratin Structure

On a tertiary structure level, keratin is characterised by the folding of its side chains and the way they interact, depending on their degree of exposure and the bonds they form with each other [68,72]. As a measure of the keratin folding state, we evaluated the shifting of the *I*_2930_ maximum position upon decomposing the corresponding high wavenumber region (HWN) broadband [53,68]. As depicted in Figure 2C, our results indicate a tendency towards lower-shifted wavenumber values for LAS than nLAS in the intermediate and bottom SC depths, with some significant differences at 50, 60, and 100% SC depth. This would indicate an increased keratin folding with less exposed side chains for LAS. Comparing directly the ratio of buried to exposed tyrosine rings in the side chains calculated as *I*_830_/*I*_850_ [71,73], we see that LAS has more buried (i.e., less exposed) aromatic rings of tyrosine than nLAS, especially in the intermediate SC depth. The corresponding curves are shown in Figure 2D, and the differences are significant for 0, 20–30, and 60% and highly significant for 40–50% SC depth. Furthermore, we compared the portion of cysteine forming disulphide bonds in keratin indicated by the *I*_690–712_/*I*_474–578_ ratio [68], which did not reveal any significant difference between LAS and nLAS (Figure 2E). Similarly, the last parameter of tertiary keratin structure we examined, namely the stability of disulphide bonds, also appears not to exhibit deviation between LAS and nLAS. The stability of disulphide bonds is given by the ratio *I*_474–508_/*I*_474–578_ [68], dividing the energetically stable *gauche*-*gauche*-*gauche*-conformation by the sum of all possible conformations (*gauche*-*gauche*-*gauche* + *gauche*-*gauche*-*trans* + *trans*-*gauche*-*trans*). As demonstrated in Figure 2F, the curves for LAS and nLAS overlap for most of the SC depth apart from the exemplary significant (but very small) differences at 80 and 100% SC depth.

### 2.5. Concentration and Mobility States of Water

**Figure 3:**
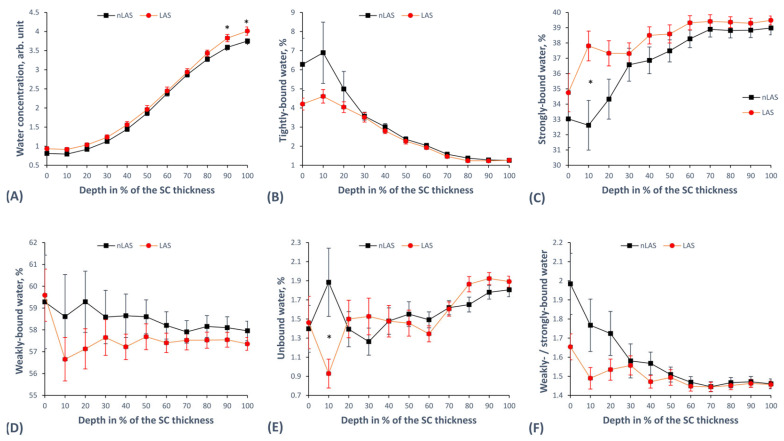
The depth-dependent water-related parameters in the SC of LAS vs. nLAS. (**A**) The water concentration calculated by the *I*_3350–3550_/*I*_2910–2965_ ratio. (**B**) The concentration of tightly bound water (DAA-bound water, band centred at 3015 cm^−1^). (**C**) The concentration of strongly bound water (DDAA-bound water, band centred at 3225 cm^−1^). (**D**) The concentration of weakly bound water (DA-bound water, band centred at 3451 cm^−1^). (**E**) The concentration of unbound water (DDA-bound and free water, band centred at 3633 cm^−1^). (**F**) The quotient *I*_3451_/*I*_3225_ representing the ratio of weakly bound to strongly bound water. The results are derived from 43 Raman profiles for LAS and 63 Raman profiles for nLAS. [*—*p* < 0.05 for significant differences between LAS and nLAS; *I*_x_—intensity at Raman peak position “x” (in cm^−1^); DA—single donor—single acceptor; DAA—single donor—double acceptor; DDA—double donor—single acceptor; DDAA—double donor—double acceptor; SC—stratum corneum; (n)LAS—non-lesional atopic skin, the error bars show the standard error of the means].

As a measure of water concentration in the SC, we examined the ratio of water to protein: *I*_3350–3550_/*I*_2910–2965_ [49], as shown in Figure 3A. It appears to be nearly the same for LAS and nLAS, apart from the bottom SC depth, where LAS seems to have a slightly higher concentration, with the difference being significant only at 90–100% SC depth.

To determine water mobility state concentrations, we decomposed the HWN spectra using 10 Gaussian functions according to previously described algorithms [67]. Next, we looked in detail at the different water mobility states according to how strongly the water bonded with its neighbouring molecules, differentiating between tightly, strongly, weakly bound, and unbound water [53,67,74]. In order to calculate the portion of tightly bound water, defined as DAA-bound water (forming single donor—double acceptor bonds with surrounding molecules), we normalised the corresponding band centred at 3015 cm^−1^ to the total water [67,75]. Figure 3B shows the results, with LAS having a lower concentration of tightly bound water at 0–20% SC depth as a trend with no statistically significant difference. Strongly bound water, defined as a double donor—double acceptor (DDAA-bound water), is associated with the band centred at 3225 cm^−1^ [67,75], and Figure 3C shows its concentration depth profile in the SC. LAS appears to have a higher concentration over the entire SC, with the strongest deviation appearing at the superficial depth and with a significant difference at only 10% SC depth. Figure 3D illustrates the concentration profile of weakly bound water, defined as a single donor—single acceptor (DA-bound water) and associated with the band centred at 3451 cm^−1^ [67,75]. Although the curves seem to differ from each other in a clear trend, with LAS having a lower concentration of weakly bound water over most of the SC depth, no difference reaches the level of statistical significance. The last type of mobility state we examined was unbound water, defined as the combination of DDA-bound water and free water and related to the band centred at 3633 cm^−1^ [67,75]. The concentration profile of unbound water is illustrated in Figure 3E and does not show a clear difference between LAS and nLAS apart from a single exemplary significant difference at the 10% SC depth. Finally, we investigated the weakly to strongly bound water ratio given by *I*_3451_/*I*_3225_, which correlates inversely with the hydrogen bonding state of water [55,67]. The resulting curves, shown in Figure 3F, represent a clear trend towards a decrease in the *I*_3451_/*I*_3225_ ratio (increase in hydrogen bonding state of water) in LAS compared with nLAS at the 0–20% SC depth, but the differences are not statistically significant.

## 3. Discussion

In this in vivo study of the atopic skin, we examined the SC molecular parameters indicating disrupted skin barrier function in LAS compared with nLAS, using noninvasive CRM.

The mean SC thickness values obtained for LAS and nLAS did not reveal any statistically significant difference. Further, the values were comparable to values obtained for HS with the same CRM method [75]. Mlitz et al. [61] were able to show a significant difference between the SC depth of nLAS and HS, with nLAS having a slightly thinner SC by a mean difference of 1 μm. This agrees with other results indicating a thinner SC with smaller corneocytes in nLAS compared with HS [76]. It should be noted that for LAS, SC thickness could be determined in approximately 20% fewer skin sites than for nLAS, which might be interpreted as an indication of a more perturbed/altered SC structure in the LAS. This is also the case to an even greater extent for lesional compared with non-lesional psoriatic skin [58].

We found the total concentration of ICL to be significantly lower in LAS than nLAS over nearly the entire SC depth (Figure 1A), in contrast to Zhang et al. [77], whose multivariate curve resolution approach failed to show this difference over the entire SC thickness, but in good agreement with the results of Janssens et al. [65]. Furthermore, a comparison with results obtained for HS using the same method [53] strongly suggests a lower ICL concentration in nLAS than HS, which would agree well with previous CRM studies [65,66]. Thus, we can confirm the conclusion of Janssens et al. [65] that ICL concentration is reduced in LAS and, to a lesser extent, in nLAS, which is a parameter of higher relevance for the SBF than the SC thickness. While FFA and cholesterol are not reduced [47], the well-established decreased amount of ceramides in atopic skin (especially ceramides 1 and 3) [6,8,9,47,78] might provide a possible explanation for the lower ICL concentration. Mechanisms leading to ceramide deficiency that have been described are reduced sphingomyelin deacylase activity [78], increased interferon-*α* [4], and increased IL-4 [16]. The reduction in ceramides has been linked with a higher TEWL value [9]. Further possible explanations that have been proposed are related to impaired lipid transport from the cells to the extracellular space [65], increased activity of SPs [9,16,31], and higher signalling of protease activator type 2 receptor leading to degraded *β*-glucocerebrosidase and acidic sphingomyelinase, important enzymes for the lipid processing [9]. The inflammation-related mechanisms are expected to play a greater role in the LAS, thus enforcing the difference between LAS and nLAS [65], as it has been shown that inflammatory cytokine concentrations are higher in LAS vs. nLAS and in nLAS vs. HS [79].

While ICL concentration provides a useful SBF parameter, the ICL organisation in the SC is even more important for maintaining an intact SBF. Our results show lower curve values for LAS than for nLAS regarding lateral organisation (Figure 1C), i.e., a shift towards a decrease in orthorhombic and an increase in hexagonal lateral packing of lipids, associated with a reduced SBF [24,25]. Comparison with HS [75] suggests that nLAS is slightly shifted towards less dense lateral packing in the superficial SC, with the values becoming comparable in deeper SC, in agreement with Verzeaux et al., who examined a similar ratio in the upper 12.5 μm of the SC in LAS and HS [66]. Using ATR-FTIR-spectroscopy, Danso et al. also confirm this finding, reporting an increased hexagonal organisation of ICL in LAS vs. nLAS and nLAS vs. HS [80]. Earlier findings indicate a reduced chain length of FFA and ceramides in atopic skin (nLAS vs. HS and LAS vs. nLAS) [33,80,81], especially the very-long-chain FFA [47,82], which causes decreased formation of the orthorhombic lateral organisation in favour of the less dense hexagonal organisation [33,83,84]. Higher levels of inflammatory cytokines (such as IFN-α) are potential factors causing this reduction in the chain length by decreasing the two elongases of fatty acids ELOVL 1 and ELOVL4 [4,80] and higher IL-4/13 levels inhibiting ELOVL3 and ELOVL6 [40]. Apart from chain length, the level of saturation in FFA also influences the lateral ICL organisation [85], with corresponding differences being observed among LAS, nLAS, and HS regarding the concentration of unsaturated FFA and the activity of the corresponding processing enzymes [80]. Danso et al. reported that the alterations in lipid-associated enzyme activity are more pronounced in LAS than in nLAS [80], which agrees with the observed differences.

The lateral ICL organisation shows an inverse relation to the lamellar (i.e., the ratio of *gauche*- to *trans*-conformation) [26]. While our results did not indicate a statistically significant difference, one may interpret as a clear trend (Figure 1B) that LAS has a lower number of *trans*-conformers (and consequently a less dense lamellar organisation of ICL) than nLAS in the region of 10–40% of the SC thickness. These depth levels are known to physiologically demonstrate the highest order of lamellar organisation in healthy skin (with the lowest ratio of *gauche*- to *trans*-conformation) [26], thus highlighting the importance of the observed difference between LAS and nLAS and indicating a reduced SBF of LAS compared with nLAS. Results from HS indicate more *gauche*-conformers in nLAS than HS [75], which is also supported by the findings of Verzeaux et al., confirming the difference between HS and nLAS [66]. In conclusion, lateral as well as lamellar ICL organisation, clearly demonstrate a reduced SBF for LAS compared with nLAS and HS.

Also relevant with respect to the ICL organisation are carotenoids without hydroxyl groups like carotenes and lycopene, which may promote the formation of orthorhombic ICL packing [27]. Apart from this important SBF-related property, carotenoids also provide valuable photoprotective and antioxidant defence against stress-induced reactive oxygen species for the SC [28]. Kake et al. were able to show that *β*-carotene improves the SBF in mice with AD [86]. Thus, the indication of a reduced concentration of carotenoids for LAS compared with nLAS (Figure 1D) would further support the thesis that LAS has a reduced SBF and suggest a factor that might partially explain the less dense lateral ICL packing in LAS. However, a physiological concentration of carotenoids in the SC of HS may vary strongly, reflecting the individual diet, lifestyle, and health status [28,69]. Thus, the carotenoid concentration could completely overlap in AD and HS patients and should be carefully compared, considering individual factors. It is known that topical and/or systemic application of carotenoids results in a long-term increase in their concentration in the HS [28]. Furthermore, topical application of antioxidant and anti-inflammatory substances has recently been shown to improve clinical symptoms in AD [87]. Therefore, it might be helpful to test in the future whether topical and/or systemic application of carotenoids in AD patients will improve the SBF and clinical disease management.

The obtained results for the NMF concentration suggest a very high significant difference between LAS and nLAS, with LAS containing less than a half of nLAS’s NMF concentration over most of the SC depth (Figure 2A) until both curves reach their lowest levels towards the deepest SC, where filaggrin is not yet broken down to the amino acids composing the NMF. The values for nLAS are in the same range as values obtained from HS determined using the same method [75], which is in disagreement with the results of Mlitz et al., who found a lower NMF concentration in nLAS than in HS, especially associated with filaggrin mutations, but also with disease severity [61]. However, the determination of NMF in that study was not depth-resolved, making it impossible to see any changes at certain SC depths. The dramatically lower NMF concentration in LAS agrees with the literature and has even been found to correlate with the disease severity, potentially providing a useful clinical marker for AD, as described by Nouwen et al. [88]. Given the important hygroscopic (primary water-binding actor in the superficial SC [68]) and antimicrobial properties of NMF, as well as its function in regulating pH and SP activity [4,16] closely linked to the AD pathophysiology, it is clear that the reduced NMF concentration in LAS implies a reduced SBF and is in good agreement with the reported lower SC hydration values obtained for LAS [34,35].

Regarding the secondary keratin structure, our results suggest a small difference in the ratio of *β*-sheets, turns, and random coils to *α*-helices, with LAS having a significantly higher ratio in the superficial SC than nLAS (Figure 2B). A possible reason might be the higher expression of proliferation- and inflammation-associated keratin types K16/17 in LAS and the reduced levels of the physiologically present K1/10 [76,89]. Totsuka et al. [90] have proposed that increased levels of T_H_2 cytokines IL-4/13 downregulate K1/10. Verzeaux et al. found an increased amount of both *α*-helices and *β*-sheets in the superficial 12.5 μm of LAS compared with HS using CRM [66]. They proposed possible explanations, which include a reduced capacity for binding water, modified filaggrin–keratin interactions, or altered IgE levels in the SC, which are known to interact with epidermal proteins with a correlation to disease severity in AD [91]. To what extent these effects play a role in the shift of the (*I*_1670_ + *I*_1685_)/*I*_1655_ ratio is not clear. A further possible interpretation is that, as the NMF concentration in LAS is drastically reduced, especially in the superficial SC depth, the keratin’s secondary structure is shifted towards a higher water-binding state as a compensation mechanism to maintain SC hydration. Results from HS [53] suggest a lower ratio for HS compared with nLAS in the bottom SC depth.

Further, we focus on the tertiary keratin structure, which is of essential importance for the mechanical structure of the corneocytes and the ability to bind water molecules, especially in the intermediate SC depth [68]. The amount of cysteine-forming-disulphide bonds and the stability of the disulphide bonds do not seem to differ between nLAS and LAS (Figure 2E,F), and also not with respect to HS as unpublished data from subjects in [75] indicates. In contrast, LAS appears to have less exposed tyrosine aromatic rings than nLAS in the intermediate SC depth (Figure 2D), implying decreased ability to bind water molecules. Further, the band position at around 2930 cm^−1^, a parameter reflecting the total amount of free side chains in tertiary keratin, is shifted lower in LAS (Figure 2C), indicating a lower number of side chains and thus less possibility to bind water. It can be speculated whether the known xerosis and lower SC hydration for atopic skin [4,34,35,77] are a cause of a more folded tertiary keratin structure or whether it is the altered keratin structure that provides a causative factor for the xerosis and lower water binding. Potential factors leading to an altered keratin structure might be inflammation [90] and reduced interaction with filaggrin or other proteins like loricrin and involucrin, which are known to be reduced in atopic skin [16,89].

The total water concentration in the LAS failed to show a significant difference with nLAS (Figure 3A), as was also the case in the recent study by Dinish et al. [63] using integrated parameters over the epidermis. Previous CRM studies report a lower total water amount in LAS compared with nLAS [77] as well as HS [61,66], in agreement with the above-mentioned facts regarding xerosis and SC hydration in AD [4,34,35,77]. The lower water content in the SC of atopic skin can be explained as a result of reduced NMF (in agreement with our results shown in Figure 2A) and, to a lesser extent, of more folded tertiary keratin [68] (Figure 2C,D). The different water mobility states we examined (Figure 3B–E) also did not reveal many significant differences between LAS and nLAS. However, a clear trend towards a lower concentration of weakly bound water (Figure 3D) and a higher concentration of strongly bound water (Figure 3C) was observed in the SC of LAS compared with nLAS. The stronger hydrogen-binding state of LAS compared with nLAS at the superficial SC depth (Figure 3F) might be attributed to the looser secondary keratin structure (Figure 2B) in LAS at the same depth. This would allow more hydrogen bonds with water molecules and might be a compensation mechanism for the corresponding much lower NMF concentration at this depth in LAS (Figure 2A). A comparison with HS data [75] reveals that LAS and nLAS have more weakly and less strongly bound water than HS, and consequently also a higher ratio of weakly to strongly bound water [67]. As weakly and strongly bound water together physiologically represent more than 90% of the total water [55,67], this shift in their corresponding ratio in atopic skin (LAS and nLAS) compared with HS can be interpreted as the lower water-binding capacity of the atopic skin. This interpretation is in agreement with the observations of Verzeaux et al. regarding functional water in the atopic skin and further points to the effects of reduced NMF and higher folding of keratin [66].

A limitation of this study is the lack of a healthy group of subjects as a direct reference and no stratification of the patients’ cohort to disease severity, filaggrin mutation status, or age of AD onset. Furthermore, although we ensured that the examined skin sites were untreated at least 24 h before measurements, most patients had received topical and systemic treatments in the past.

## 4. Materials and Methods

### 4.1. Confocal Raman Microspectroscopy (CRM)

The CRM measurements were conducted with the “skin composition analyzer” appropriate for in vivo/ex vivo measurements (RiverD International B.V., Model 3510, Rotterdam, The Netherlands). In order to obtain skin spectra in the fingerprint region (FP, 400–2000 cm^−1^) and in the HWN (2000–4000 cm^−1^), a 785 nm laser (20 mW on the skin surface with 5 s exposure time) and a 671 nm laser (17 mW on the skin surface with 1 s exposure time) were used. Starting from above the skin surface, Raman profiles were recorded in increments of 2 μm, reaching a total depth of 40 μm. The depth resolution was ≤5 µm, and the spectral resolution was 2 cm^−1^. For each profile, FP and HWN spectra were recorded in precisely the same position and depth with the help of the CRM’s piezoelectric actuator (PIFOC piezo flexure nanopositioner, Physik Instrumente GmbH & Co. KG, Waldbronn, Germany). The time to obtain a complete FP + HWN depth profile of a single skin site was approximately 2 min. For more details on the CRM method utilised, refer to the literature [49,92]. To determine the NMF depth profiles, we followed the same method described by Caspers et al. [49], using the Skin Tools 2.0 (RiverD International B.V., Rotterdam, The Netherlands) software.

### 4.2. Subjects and Measurements

**Figure 4:**
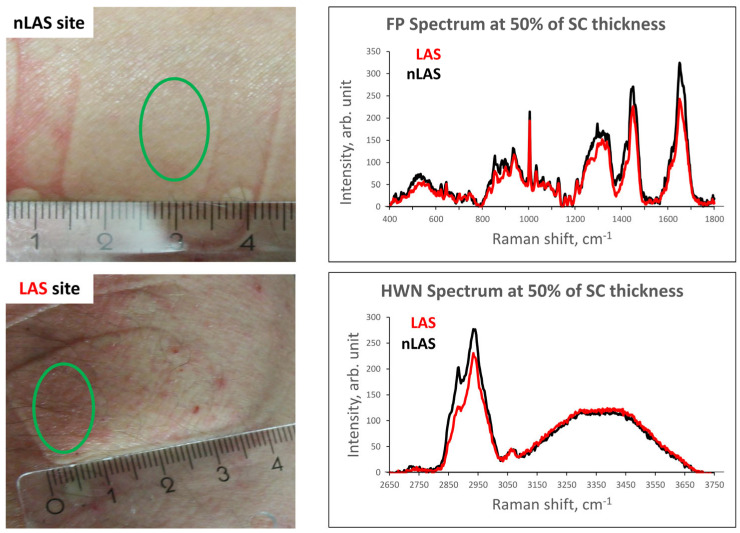
One exemplary nLAS and one LAS site with green circles designating the exact areas examined in vivo using CRM (**left**). The corresponding exemplary Raman profiles of the skin sites are shown for the depth level of approx. 50% of the SC thickness (≈12 µm), one for LAS and one for nLAS, in the FP and HWN regions (**right**). [SC—stratum corneum; (n)LAS—non-lesional atopic skin; FP—fingerprint region; HWN—high wavenumber region].

In total, 21 patients diagnosed with AD (9 female and 12 male), with skin type I–III in the Fitzpatrick classification [93] and a mean age of 34 ± 10 years, were recruited prospectively for this study (Supplementary Table S1). The age of AD onset among the patients varied from infancy (aged 0–2: 13) and childhood (aged 3–13: 7) to puberty/adulthood (aged ≥14: 1). All patients had at least one active AD lesion at the time and were asked to keep the body parts (forearm and elbow) intended for measurements free of any topical treatments at least 24 h before the CRM measurements to exclude a possible influence of the formulation on the molecular structure of the SC [75]. Measurements were conducted for each patient at healthy-appearing, non-lesional skin sites (corresponding to nLAS) and eczematous lesional skin sites (corresponding to LAS) in vivo; no biopsies were taken. Each complete measurement, including the FP and corresponding HWN spectra, was conducted at a distinct skin site. The atopic lesions were chosen with none-to-mild lichenification (Table 1 and Supplementary Table S3), evaluated by a local EASI (Eczema Area and Severity Index) score [94], and photo-documented at the time of examination. Two exemplary skin sites (one for nLAS and one for LAS) and their corresponding FP and HWN spectra at an exemplary depth level are shown in Figure 4. No manipulation of the lesions in any form took place prior to the measurements. The laboratory conditions were standardised (temperature ≈ 20 °C), and the acclimatisation time was not shorter than 15 min. All patients were informed in detail about the study and provided written consent. The ethics committee of the Charité—Universitätsmedizin approved the study (EA1/145/17), and all procedures complied with the Declaration of Helsinki revised in 2013.

### 4.3. Data Processing and Statistical Analysis

Profiles of low quality were excluded from further analysis, i.e., in case of contact instability or movement during the measurement, noncompliance with the no-pretreatment requirement 24 h before measurement, or ambiguity between nLAS or LAS in the examined skin site (Supplementary Table S1). The remaining CRM profiles were recorded from 18 LAS sites (yielding 71 profiles) and 13 nLAS sites (yielding 78). After preprocessing the recorded Raman spectra and subtracting the fluorescence background, following the methods established in our group before [53,67,68], the SC boundaries were determined by defining its surface position (0% SC depth) as the point where the keratin-related band at 1655 cm^−1^ reaches the half of its maximal intensity [92], and the bottom of the SC (100% SC depth) as the point where the first derivative of the water concentration profile curve reaches 0.5 [95]. Every examined parameter was interpolated from 0 to 100% of the SC depth with increments of 10%. In accordance with the literature [53,55,58,68], *t*-tests were used to obtain the significance level of the comparison of the means for the parameters of LAS and nLAS, predefining differences with *p* < 0.05 as significant (*) and *p* < 0.01 as highly significant (**). The programs used for analysing the data were Skin Tools 2.0 (RiverD International B.V., Rotterdam, The Netherlands), Matlab R2019b (The MathWorks Inc., Natick, MA, USA), Origin 2020b (OriginLab Corporation, Northampton, MA, USA), and Excel 2016 (Microsoft Corporation, Redmont, WA, USA).

## 5. Conclusions

In summary, the results of the conducted study strongly suggest a reduced SBF for LAS compared with nLAS, as LAS has a lower concentration of NMF, a lower concentration and less dense lateral organisation of ICL, a reduced concentration of carotenoids, and an altered secondary and tertiary keratin structure, allowing less water binding. Comparisons with other studies using the same methods applied in HS seem to confirm the intermediate position of nLAS between LAS and HS regarding its SBF. However, to the best of our knowledge, this is the first time that the entire SC of LAS and nLAS are compared using CRM in a depth-dependent manner regarding molecular SBF-defining composition and parameters. Comprehending the alterations of the atopic SC and its SBF in detail is not only essential in understanding the pathogenetic aetiology of the disease but also in developing targeted therapies, as well as optimal galenic formulations for LAS and nLAS. Furthermore, the current study shows that the performed CRM and analytical methods are useful for optimal remission assessment and treatment control in future interventional studies in AD.

## Figures and Tables

**Table 1:** Descriptive statistics of the local EASI parameters of the atopic lesions used for the analysis (compare also Supplementary Table S3).

Local EASI	Erythema	Oedema/Papulation	Excoriation	Lichenification
median	2	1	0.5	0
maximum	3	1	3	1
minimum	1	0	0	0

## Data Availability

Data can be shared upon request from the corresponding authors.

## Supplementary Materials

The supporting information can be downloaded at: https://www.mdpi.com/article/10.3390/ijms241914636/s1.

## Abbreviations

This section contains a list of abbreviations used in the main text and Supplementary Material.

AD	atopic dermatitis
CRM	confocal Raman microspectroscopy
DA	single donor—single acceptor
DAA	single donor—double acceptor
DDA	double donor—single acceptor
DDAA	double donor—double acceptor
EASI	eczema area and severity index
FFA(s)	free fatty acids
FP	fingerprint region
HWN	high wavenumber region
HS	healthy skin, i.e., skin of healthy subjects
*I*_x_	intensity at Raman peak position “x” (in cm^−1^)
IL	interleukin
ICL(s)	intercellular lipid(s)
Kx	keratin type “x”
LAS	lesional atopic skin
nLAS	non-lesional atopic skin
NMF(s)	natural moisturizing factor(s)
SC	stratum corneum
SBF	skin barrier function
SG	stratum granulosum
SP	serine protease(s)
TEWL	transepidermal water loss
T_H_2	type 2 T-helper cells
