# Supplementary material for: Atopic Dermatitis: Molecular Alterations between Lesional and Non-Lesional Skin Determined Noninvasively by In Vivo Confocal Raman Microspectroscopy
Source: Int J Mol Sci. 2023 Sep 27;24(19):14636. doi: 10.3390/ijms241914636 (PMC10572245; doi:10.3390/ijms241914636)
Supplement: Supplementary file 1 [file ijms-24-14636-s001.zip › ijms-2552967-supplementary.pdf]

Supplementary Materials

Atopic Dermatitis: Molecular Alterations between Lesional and Non-Lesional Skin Determined Noninvasively by In Vivo Confocal Raman Microspectroscopy

Michael Zolotas [1], Johannes Schleusener [1,*], Jürgen Lademann [1], Martina C. Meinke [1], Georgios Kokolakis [2] and Maxim E. Darvin [1,*]

[1] Center of Experimental and Applied Cutaneous Physiology, Department of Dermatology, Venereology and Allergology, Charité – Universitätsmedizin Berlin, Corporate Member of Freie Universität Berlin and Humboldt-Universität zu Berlin, Charitéplatz 1, 10117 Berlin, Germany

[2] Psoriasis Research and Treatment Centre, Department of Dermatology, Venereology and Allergology, Charité – Universitätsmedizin Berlin, Corporate Member of Freie Universität Berlin and Humboldt-Universität zu Berlin, Charitéplatz 1, 10117 Berlin, Germany

* Correspondence: johannes.schleusener@charite.de (J.S.); maxim.darvin@protonmail.com (M.E.D.)

Supplementary Table S1. Demographics and lesions of all recruited AD patients (AD: atopic dermatitis; F: female, M: male; I: infancy (aged 0–2), C: childhood (aged 3–13), PA: puberty/adulthood (aged ≥14); EASI: Eczema Area and Severity Index (4 numbers from left to right: erythema, oedema/papulation, excoriation and lichenification of the lesion); (n)LAS: (non)lesional atopic skin).

Patient-ID	Age	Sex	AD age onset	Skin Type	Number of lesions	Local EASI score	LAS profiles	nLAS profiles	Excluded profiles with reason of exclusion
**1**	37	M	I	II	1	1 1 0 1	6	6	LAS: ambiguity between LAS/nLAS
**2**	31	M	I	II	1	2 1 1 0	6	6	LAS: ambiguity between LAS/nLAS
**3**	53	M	C	II	1	2 1 1 0	6	6	
**4**	38	F	I	II	2	2 1 1 0 / 1 1 0 0	4 / 2	6	
**5**	31	F	PA	II	1	1 1 0 0	6	6	
**6**	48	F	C	II	2	2 0 0 0 / 1 0 0 0	3 / 3	6	LAS + nLAS: use of cortisone prior to measurement
**7**	28	F	I	II	2	2 1 3 0 / 2 1 2 0	5 / 1	6	
**8**	55	M	I	III	2	1 1 0 0 / 2 1 0 0	4 / 2	6	LAS + nLAS: use of cortisone prior to measurement
**9**	20	M	I	II	2	2 1 2 1 / 3 1 0 0	3 / 3	6	
**10**	46	M	I	II	2	2 0 0 0 / 2 0 0 0	3 / 3	6	
**11**	29	M	C	II	1	2 1 1 0	6	6	
**12**	22	M	C	II	3	2 1 2 0 / 1 0 0 0 / 2 1 1 0	3 / 3 / 2	6	LAS + nLAS: instability/movement during measurement
**13**	29	M	I	III	1	1 0 0 0	6	6	LAS + nLAS: ambiguity between LAS/nLAS
**14**	40	M	C	III	2	2 1 1 1	3	6	nLAS: ambiguity between nLAS/LAS
**15**	31	M	I	II	2	1 0 1 0 / 0 1 0 1	3 / 3	6	LAS + nLAS: use of cortisone prior to measurement
**16**	37	F	C	III	2	2 0 0 0 / 2 2 1 1	4 / 4	6	LAS + nLAS: instability/movement during measurement
**17**	35	F	I	III	1	1 1 0 0	6	6	LAS: instability/movement during measurement
**18**	32	F	I	III	2	2 1 0 0 / 1 1 0 0	6 / 1	6	
**19**	33	F	I	III	1	2 1 0 0	6	6	nLAS: ambiguity between nLAS/LAS
**20**	23	F	C	III	2	1 0 1 0 / 2 1 0 1	3 / 4	6	
**21**	23	M	I	III	1	2 1 1 1	6	6	

Supplementary Table S2. Demographics and lesions of the AD patients after exclusion of inadequate profiles indicating successful SC-Th determination (AD: atopic dermatitis, SC-Th: stratum corneum thickness; F: female, M: male; I: infancy (aged 0–2), C: childhood (aged 3–13), PA: puberty/adulthood (aged ≥14); EASI: Eczema Area and Severity Index (4 numbers from left to right: erythema, oedema/papulation, excoriation and lichenification of the lesion); (n)LAS: (non)lesional atopic skin).

Patient-ID	Age	Sex	AD age onset	Skin Type	Number of lesions	Local EASI score	LAS profiles	nLAS profiles	LAS with SC-Th	nLAS with SC-Th
**1**	37	M	I	II	0		0	6	0	2
**2**	31	M	I	II	0		0	6	0	4
**3**	53	M	C	II	1	2 1 1 0	6	6	4	3
**4**	38	F	I	II	2	2 1 1 0 / 1 1 0 0	4 / 2	6	2 / 2	4
**5**	31	F	PA	II	1	1 1 0 0	6	6	6	6
**6**	28	F	I	II	2	2 1 3 0 / 2 1 2 0	5 / 1	6	2 / 1	6
**7**	20	M	I	II	2	2 1 2 1 / 3 1 0 0	3 / 3	6	0 / 3	4
**8**	46	M	I	II	2	2 0 0 0 / 2 0 0 0	3 / 3	6	2 / 1	6
**9**	29	M	C	II	1	2 1 1 0	6	6	2	6
**10**	40	M	C	III	1	2 1 1 1	3	0	0	0
**11**	35	F	I	III	0		0	6	0	5
**12**	32	F	I	III	2	2 1 0 0 / 1 1 0 0	6 / 1	6	4 / 0	6
**13**	33	F	I	III	1	2 1 0 0	6	0	5	0
**14**	23	F	C	III	2	1 0 1 0 / 2 1 0 1	3 / 4	6	1 / 4	6
**15**	23	M	I	III	1	2 1 1 1	6	6	4	5

Supplementary Table S3. The detailed local EASI (Eczema Area and Severity Index) parameters of each atopic lesion used for the analysis after exclusion.

Lesion number	Erythema	Oedema/Papulation	Excoriation	Lichenification	Sum
**1**	2	1	1	0	4
**2**	2	1	1	0	4
**3**	1	1	0	0	2
**4**	1	1	0	0	2
**5**	2	1	3	0	6
**6**	2	1	2	0	5
**7**	2	1	2	1	6
**8**	3	1	0	0	4
**9**	2	0	0	0	2
**10**	2	0	0	0	2
**11**	2	1	1	0	4
**12**	2	1	1	1	5
**13**	2	1	0	0	3
**14**	1	1	0	0	2
**15**	2	1	0	0	3
**16**	1	0	1	0	2
**17**	2	1	0	1	4
**18**	2	1	1	1	5
